# The Roles of Fibroblast Growth Factors in the Testicular Development and Tumor

**DOI:** 10.1155/2013/489095

**Published:** 2013-09-18

**Authors:** Xin Jiang, Melissa Skibba, Chi Zhang, Yi Tan, Ying Xin, Yaqin Qu

**Affiliations:** ^1^The First Hospital of Jilin University, Changchun 130021, China; ^2^KCHRI at the Department of Pediatrics, The University of Louisville, Louisville 40202, USA; ^3^The Chinese-American Research Institute for Diabetic Complications, Wenzhou 325200, China; ^4^Key Laboratory of Pathobiology, Ministry of Education, Jilin University, 126 Xinmin Street, Changchun 130021, China

## Abstract

Fibroblast growth factors (FGFs) are classically known as hormonal factors and recent studies have revealed that FGFs have a key role in regulating growth and development of several reproductive organs, including the testis. The testis is mainly consisted of germ cells, Sertoli cells and Leydig cells to develop and maintain the male phenotype and reproduction. This review summarizes the structure and fuctions of testis, the roles of FGFs on testicular development and potential involvement in testicular tumor and its regulatory mechanism. Among 23 members of FGFs, the FGF-1, FGF-2, FGF-4, FGF-8, FGF-9, and FGF-21 were involved and describe in details. Understanding the roles and mechanism of FGFs is the foundation to modeling testicular development and treatments in testicular disease. Therefore, in the last part, the potential therapy with FGFs for the testis of cancer and diabetes was also discussed.

## 1. Introduction

The adult mammalian testis is the important organ within the male reproductive system and has two major physiological functions: the production of spermatozoa via spermatogenesis and testosterone (TE) via steroidogenesis [1]. Development and maintenance of the male function of fertility are all dependent on the activity of the germ cells, Sertoli cells, and Leydig cells of the testis. Germ cells are one of the two types of cells in the body and the other is somatic cells. During the male development, germ cells originate from the gut of the embryo and migrate to the developing gonads. Then they can undergo mitosis or meiosis and differentiate into sperm [2, 3], which fuses with oocytes during fertilization. Sertoli cells, known as the nursery cell, affect and support germ cells by anchoring junctions in seminiferous epithelium. Leydig cells secrete testosterone to stimulate Sertoli cell activity and germ cell proliferation (spermatogenesis) [4]. Germ cells are also affected by follicle-stimulating hormone (FSH) that is produced from the pituitary gland and is a key regulator in the production and function of testosterone.

Beside being regulated by endocrine factors, function of the testis are also mediated by paracrine pathway, including hormones, growth factors, and cytokines [5–7]. Growth factors, such as fibroblast growth factors (FGFs), are important hormone-related substances that promote cell proliferation, regulate tissue differentiation, and modulate organogenesis [8]. The FGF family consists of 23 members and have been localized in some kind of cells of the male reproductive tract and have shown to be important in regulating the growth and the development of several reproductive organs, including the testis [9, 10]. The regulation of testicular cell growth is required for the maintenance of spermatogenesis in the adult testis. On the other hand, testicular apoptosis, as programmed cell death, also plays an important role in controlling the number of male germ cells and eliminating defective germ cells during testicular development and spermatogenesis as well as preventing testicular tumor. Testicular apoptosis is controlled by hormones and FGFs [11–13]. The review article will outline the role of FGFs on testicular development, proliferation and apoptosis in normal and pathological conditions, and the regulation of testicular tumor.

## 2. FGFs Signaling

FGFs are hormonal factors that provide a range of determining and regulating functions in some tissues or organs [14, 15]. Although FGFs were first discovered as a mitogen on 3T3 fibroblasts, only some members of the FGF family promote growth and strictly act on fibroblasts [2]. Among the 23 members of FGF family, seven subfamilies are further divided based on phylogeny, common structural characteristics, and sequence identity rather than on functional similarity [16]. FGF plays an important role in many processes including development, morphogenesis, angiogenesis, hematopoiesis, cell proliferation, differentiation, survival, and migration [17–19].

The biological significance of FGF signaling system for human health and development is illustrated in recent observations [20]. The correct maintenance and regulation of FGF signaling is evident from human and mouse genetic studies, which showed a variety of developmental disorders including dominant skeletal diseases, infertility, and cancer if some signaling mutations lead to the disruption of FGFs [10, 21, 22].

Accumulating evidence indicates the role of FGFs as a critical regulator for long-term energy balance and metabolism. In particular, the endocrine-acting FGF-19 subfamily including FGF-19, FGF-21, and FGF-23 was shown to be involved in glucose, lipid, bile acid, phosphate, and vitamin D metabolism; however, the mechanisms underlying their functions as metabolic regulators still need to be defined [13, 23, 24].

Numerous studies have focused on the expression and the role of FGFs, including FGF-1 (aFGF), FGF-2 (bFGF), FGF-4, FGF-5, FGF-8, FGF-9, FGF-13, FGF-18, and FGF-21 in the testis [8, 10, 13, 25–27]. Moreover, the role of FGFs is different and it will be involved in development, cell proliferation, differentiation, and apoptosis in the testis.

## 3. Testicular Structure

The adult mammalian testis is a vital organ within the scrotum of the male reproductive system and has two major physiological functions as mentioned above [1]. The surface of testis has a transparent layer of dense connective tissue, named testicular tunica albuginea, providing a protective barrier on the testis. The testicular tunica albuginea thicken and extend into the testis which will be separated into many small testis lobules, filled with testicular essence [28, 29]. Testicular essence is mainly composed of seminiferous tubules, which are the functional units that produce spermatozoa during spermatogenesis. Regulation is done largely by FSH and testosterone produced from the pituitary gland and Leydig cells, respectively.

Spermatogenesis takes place in the seminiferous tubule, whereas steroidogenesis occurs in Leydig cells found in the interstitium [30]. Each seminiferous tubule is composed of the seminiferous tubule lumen and seminiferous epithelium which is constituted by Sertoli cells and germ cells at different stages of development: spermatogonia, primary spermatocyte, secondary spermatocyte, pachytene spermatocytes, round spermatids, elongating spermatids, and spermatozoon. Between these tubules is the interstitium, containing the androgen producing Leydig cells, red blood cells, and a blood vessel [1, 31]. The mature spermatozoa detaches from the epithelium in the center of seminiferous tubule in the process of sperm release, known as spermiation. Development and maintenance of the male phenotype and establishment of fertility are all dependent upon the activity of the Sertoli cells and Leydig cells of the testis. Sertoli cell, also known as the nursery cell, supports as many as 50 germ cells in the epithelium and plays a crucial role in germ cell maturation. Sertoli cells are associated with germ cells by different anchoring junctions with the basement membrane, such as cell to cell adherent junctions during different stages of their development [1]. In essence, Sertoli and germ cells, particularly spermatogonia, are resting on the basement membrane at different stages of the seminiferous epithelial cycle, relying on its structural and hormonal support [32]. Leydig cells are dependent on LH and FSH, and Sertoli cell can also affect Leydig cell function. Testosterone secreted by the Leydig cells acts with FSH to stimulate Sertoli cell activity and spermatogenesis [33].

## 4. FGFs and Testicular Development

FGFs are classically considered to be paracrine factors and are known for their roles in tissue patterning and organogenesis during embryogenesis. FGFs have been localized in many cells of the male reproductive tract and have been shown to be important in the regulation for the growth and development of several reproductive organs including the testis [8, 34, 35]. Studies have shown that FGF-9 signaling can stimulate mesenchymal cell proliferation and migration of mesonephric cells into the testis during development, contributing to the formation of the interstitial compartment of the testis [36].

In mammals, there is a poleward expansion of testiculogenic programs along the anteroposterior axis of developing XY gonads. Sry (sex determining region of Chr Y) and its target gene, Sox9, are essential in the supporting cells for initiating Sertoli cell differentiation and testis formation [37, 38]. Sry and Sox9 alone cannot drive center-to-pole expansion of testiculogenic programs at the initial phase of testis differentiation. However, FGF-9 signaling is crucial for the maintenance of Sry and Sox9 expression, and FGF-9 can act as a diffusible conductor for the poleward expansion of tubulogenic programs and testis cord formation in developing XY gonads [39]. 

Also, the role of FGF-9 on testis development is relative with Wnt4, which can act as an antagonistic signal against FGF-9 signaling in developing XY gonads [39]. The supportive role of the central domain could be substituted by exogenous FGF-9 supply, whereas reduction of Wnt4 activity did not rescue the tubulogenesis defect in the pole segments [10]. FGF-9 is also critical to a repression program in the XY gonad that blocks the female gene expression that would otherwise antagonize male development. Jameson et al. used Fgfr-2/Wnt4 and Fgf-9/Wnt4 double mutant mice to show that FGF signaling promotes male sex determination by repressing the female-promoting genes. Male development would abort if the male signaling was unable to repress Wnt4 due to loss of either FGF-9 or FGFR-2 [25].

Interestingly, another study has identified Fgf-13 and Fgf-18 as new candidate genes for the effect on testes differentiation and pre-Sertoli cell function; however, the exact mechanism of the involvement requires further investigation [40]. Fgf-14 and Fgf-10 mRNA were also shown to be predominantly expressed in mouse brain and testis by RNA protection using a probe for Fgf-14 mRNA, but their role in the testis has never been addressed [41, 42].

## 5. FGFs and Their Effect on Testicular Leydig and Sertoli Cells

FGFs have been localized to many cells throughout the testis, including Sertoli, Leydig, and germ cells.* In vitro*, FGFs as a potent mitogenic factor stimulate both DNA synthesis and cell multiplication of cultured pig Sertoli cells and also their phenotypic expression [43]. FGF-2 was found to significantly increase the number of Sertoli cells after culturing the cells for 3 to 6 days in isolated fetal, newborn, or 3-day-old rats. FGF-2 did not increase the [^3^H] thymidine labeling index of Sertoli cells, indicating that FGF-2 was a survival factor for these cells *in vitro *[44].

As a mitogenic factor, FGF-2 might also be involved in the development of the immature testis. Indeed, immunohistochemical evidence indicates the presence of FGF-2 in fetal Leydig cells [45] and mature Leydig cells [46]. The effect and mechanism of FGF-2 on testicular steroidogenesis were investigated using cultures of purified porcine Leydig cells from immature intact animals as a basis for a primary model. FGF-2 increased basal and human chorionic gonadotropin (hCG)-induced testosterone accumulation in the medium following a long-term treatment in cultured Leydig cells. FGF-2 also affected the maximal steroidogenic capacity of the Leydig cells strongly suggest a pleiotropic role [47].

 It is evident that locally produced factors are also involved in the regulation of Leydig cell function. This includes members of the FGF family which have been implicated in the development of the rat testis [9]. Laslett et al. examined the effects of FGF-1 and FGF-2 on Leydig cell steroidogenesis by cells from 5-, 21-, and 90-day-old rats. They found that both of FGF-1 and FGF-2 had stimulatory effects on basal LH-stimulated testosterone production by fetal Leydig cells and basal 5*α*-androstane-3*α*,17*β*-diol production by immature Leydig cells. There was no effect of FGF-1 and FGF-2 on basal testosterone production by adult Leydig cells; however, FGF1 alone inhibited LH-stimulated testosterone production by adult Leydig cells in a dose-dependent manner. This data demonstrated that the effects of FGF-1 and FGF-2 are dependent on the specific stage of Leydig cell differentiation and development and may vary accordingly at different stages of development [48].

FGF-8 is widely expressed in embryonic tissues and apparently has a specific function in the elongation of the body axis, morphogenesis of the central nervous system, limb, and face. However, in adult mouse, Fgf-8 mRNA expression has been detected by Northern blotting only in the testis [49]. The Sertoli cells of testis are mitotically active in the fetal testis at the time when the accumulation of Fgf-8 hybridization signal in the prespermatogonia was observed [50]. Accordingly, a very low level of FGF-8 expression in adult testis could be expected since the proliferation of murine Sertoli cells decreases markedly after birth and ceases during puberty [51]. The timing of FGF-8 expression suggests that it has a specific function in the maturation of the seminiferous epithelium of testis [52].

## 6. FGFs and Their Effects on Testicular Germ Cells

### 6.1. Proliferation and Differentiation

FGFs have regulated a broad range of cellular activities, including cell proliferation and differentiation in many organs during embryonic development [53]. Fully functional sperm cannot develop independently and rely heavily upon the unique environment provided by the testis. The regulation of testicular cell growth is essential in the developing testis and is required for the maintenance of spermatogenesis in the adult testis. The rapid rate of germinal cell proliferation and the continuation requires the presence of specific growth factors in the adult testis [54].

Some FGF members are also involved in the paracrine regulation of testis germ cell growth. Supplement of FGF-2 significantly increased the number of gonocytes separated from either newborn or 3-day-old animals and cultured for 6 days. It was found that germ cells doubled in those cultures compared to those in the control cultures. Treatment with a neutralizing antibody against FGF-2 to control cultures caused a significant decrease in the number of gonocytes compared to that in untreated cultures. FGF-2 significantly stimulated the proliferative activity of the gonocytes, indicating that FGF-2 is involved in the survival as well as a mitogenic factor for these cells. So, these data suggested that FGF-2 was an important factor at the start of spermatogenesis [44].

FGF-9 signaling has also been shown to stimulate proliferation of mesenchymal cells and migration of mesonephric cells into the testis during development, contributing to the formation of the interstitial compartment of the testis [36]. It has been observed that FGFs stimulated mitotic proliferation of primitive spermatogonia and spermatogenic meiosis through tyrosine kinase receptors (FGFRs) partially to enhance classical mitogen activated protein kinase (MAPK) activity [55].

There was a lack of reports regarding FGFs directly in regulation on cell proliferation and differentiation in the testicular germ cells, so there will be a need for further study and discussion in this field.

### 6.2. Apoptosis

Apoptosis, or programmed cell death, is essential for the development and homeostasis in multicellular organisms. Apoptosis also plays an important role in controlling the number of male germ cells and eliminating defective germ cells during testicular development and spermatogenesis [56]. In recent years, more and more evidence reported that ligands of FGF-1, -4, -8, -10, and -14 were expressed in mouse or rat testes; however, only FGF-4 and FGF-21 showed the directly antiapoptotic effects on germ cells, including on some disease conditions [41, 42, 57].

In the present study, the Fgf-4 gene was expressed in the testicular cells of adult mouse testes and may offer insights into the biological significance in testicular functions [27]. Yamamoto et al. demonstrated for the first time that the Fgf-4 gene in the testis resulted in markedly enhanced spermatogenesis and acts as a survival factor for germ cells [58]. To further investigate the function and therapeutic potency of FGF-4, transgenic mice with enhanced FGF-4 expression in the testis were treated with adriamycin (ADR), which is an anticancer drug causing severe testicular toxicity. The result indicated that induced expression of FGF-4 markedly enhanced the recovery of ADR-induced testicular damage and adenoviruses carrying the Fgf-4 gene ameliorated testicular toxicity of ADR.

Besides, as an important survival factor, FGF-4 protects neural cells from nitric oxide-induced apoptosis [59]. Also, FGF-4 inhibited apoptosis in the dental mesenchyme when applied locally, suggesting the involvement of this gene in the prevention of apoptosis in dental tissues *in vivo *[60]. To investigate the potential role of FGF-4 as an antiapoptotic agent in the testes of adult mice, the overexpressed FGF-4 was exposed to mild hyperthermia inducing germ cell apoptosis. Then the testicular cell apoptosis was checked by TUNEL staining. The results indicated that FGF-4 significantly reduced the apoptosis of germ cells, and the expression of FGF-4 in the testes was upregulated *in vivo* when the testes are exposed to heat stress [61]. Thereby, FGF-4 could be an important factor for spermatogenesis and present a new paradigm to treat impaired fertility. However, the exact mechanism of FGF-4 on antiapoptosis in the testis remains unclear.

Because some studies demonstrated that FGFs could protect other tissues or cells from apoptosis though MAPK signaling pathway [8, 61], the classical MAPK phosphorylation by FGF-4 was evaluated in purified germ cells *in vitro* to examine the role of MAPK pathway in antiapoptotic effects of FGF-4 on germ cells. Their results showed that FGF-4 induced the phosphorylation of ERK1/2 in both Sertoli and germ cells after stimulation. Thus, FGF-4 could trigger the ERK/MAPK signaling pathway, which appears to induce antiapoptotic effects in germ cells [61].

FGF-21 is a novel member of the FGF family identified by Nishimura et al. [62]. Some evidence indicates FGF-21 as a critical regulator of long-term energy balance and metabolism. Also there are some reports that FGF-21 improved the survival of pancreatic â-cells [63] and oxidized low density lipoprotein- (ox-LDL) provided protection for FGF-21 from apoptotic cell death in cardiac microvascular endothelial cells [64]. In the testis, the expression of Fgf-21 mRNA was founded [65], but the biological function of FGF-21 in the testis remains unclear.

Our study showed that the deletion of Fgf-21 gene does not affect testicular cell proliferation, but significantly increases the spontaneous incidence of testicular apoptosis accompanied with the increased ratio of Bax/Bcl2 expression. It means that FGF-21 is involved in the regulation of testicular germ cells apoptosis through mitochondrial-dependent apoptosis pathway.

Testicular apoptotic cell death occurs in many conditions, including the normal spermatogenesis and also chronic diseases such as diabetes. Diabetes induces testicular apoptotic cell death predominantly through mitochondrial and endoplasmic reticulum (ER) stress associated cell death pathways, which may be metabolic abnormality induced oxidative damage [66, 67]. Deletion of Fgf-21 gene significantly enhances diabetes-induced testicular apoptosis along with the activation of mitochondrial-dependent apoptosis pathways, endoplasmic reticulum stress-dependent pathways, and oxidative damage but did not change the expression of cleaved caspase-3 and caspase-8, which was significantly prevented by the supplementation of exogenous FGF-21. These results suggest that Fgf-21 gene may be involved in maintaining normal spermatogenesis and also protect the germ cells from diabetes-induced apoptotic cell death probably via the prevention of diabetes-induced oxidative damage or maintaining the functions of mitochondria and endoplasmic reticulum [13].

Meanwhile, some studies have demonstrated that other FGF family members such as FGF-1 and FGF-2 have the similar antioxidative function [63, 64, 68]. However, the exact underlying mechanism of FGFs to decreasing oxidative stress and maintenance of mitochondrial function is not clear, and further studies are needed to explain it. Further analysis of other genes of the FGF family in the testes can be performed to get a better understanding of the germ cells network.

## 7. FGFs and Testicular Tumor

The incidence of malignant tumors of the testis is increasing rapidly in industrialized countries. Even the etiology of testicular cancer remains unknown, both congenital and acquired factors have been associated with tumor development. Due to FGFs being involved in embryonic testis development and differentiation, a few studies have attempted to elucidate the role of FGFs in testicular cancer [69, 70]. FGFs may promote testicular growth and metastasis by different mechanisms, mainly as the mitogens for tumor cells, induction of matrix metalloproteinases (MMPs), and angiogenesis.

Early studies suggested that FGF-4 was overexpressed in different germ cell tumors [71, 72]. Strohmeyer et al. found significant association between FGF-4 and tumor stage in non-seminomas, that is, some local tumors, tumors with nodal or distant metastasis showing an overexpression of FGF-4 [71]. Another immunohistochemical study on primary testicular germ cell tumors showed predominant expression of FGF-4, FGF-8, and FGFR1 in non-seminomatous and highly proliferative components of the tumors. These results suggest that FGF-4 and FGF-8 are both involved in testicular cancer [70].

The patients with seminoma or nonseminoma showed a significant elevation of FGF-2 either at the serum level and/or in the tumor tissue expression. Analyzing human teratocarcinoma cells *in vitro* also showed that low concentrations of FGF-2 stimulated their proliferation, whereas higher concentrations induced cell migration [73]. Other studies with other kinds of tumors showed that there was an autocrine FGF-2 activation loop to control ERK1/2 activation and then FGF2 downregulates E-cadherin expression to induce cell invasion through activation of PI3K/AKT/mTOR and MAPK/ERK signaling [74, 75].

Beside the directly regulating mitosis and migration of tumor cells, FGFs also showed the lymphangiogenic and angiogenic effects. FGF-2 stimulated proliferation and migration of lymphatic and vascular endothelial cells, which is associated with AKT/mTOR/p70(S6 kinase) and MAPK/ERK pathway activation and intracellular Ras-JNK signaling down-regulation [76–78].

Even though there is no direct evidence to prove that FGFs affect testicular tumor metastasis through regulating MMPs, a few researches have showed the connection between FGF family and MMP members. For example, FGF-1 initiated the endothelial cell migration through activating MMP-1 to facilitate angiogenesis [79]. FGF-1 also induces MMP-9 expression in breast cancer cells to accelerate tumor invasion and metastasis [80]. These studies suggested that mTOR and MAPK/ERK pathways and MMP members may all involve in directly regulating FGFs on testicular tumor, which needs further studies to be proved.

## 8. FGFs Potential Therapy in Clinics

Because FGFs are involved in the complex signaling network in both normal and malignant testis tissues, they represent potential diagnostic markers and/or targets for therapy of testicular sterility and cancer. FGF-2 was elevated in the tissue and serum of patients with testicular cancer, including the seminomatous as well as nonseminomatous tumors. Therefore, FGF-2 may represent a promising new diagnostic marker for testicular cancer with high sensitivity [81].

Meanwhile, FGFs, as proangiogenic cytokines, may facilitate angiogenesis to promote cancer development through binding with their receptors, FGFR1-4. Correspondingly, a few drugs targeting the FGF ligand-FGF receptor interaction are promising for the treatment of cancer, including testicular cancer. FGFR directed therapeutics are subcategorized as either selective FGFR inhibitors or multitargeted tyrosine kinase inhibitors [82]. For example, Brivanib is an oral and potent nonselective inhibitor of FGF/VEGF and now has been used in the late-phase clinical trials [83].

On the other hand, the neovascularization function of FGFs will provide effective therapy to ischemia disease. For example, utilization of sustained release system of FGF-2 has shown sufficient neovascularization in diabetic limb ischemia. The method may provide a more effective therapeutic angiogenesis in patients with diabetes. Clinical trial of therapeutic angiogenesis for severe hindlimb ischemia has already started [84]. Supplements of FGFs have a potential benefit in protecting testicular ischemia-reperfusion injury [85].

## 9. Summary

FGF signaling is critical for a broad range of determining and regulating functions including development, angiogenesis, hematopoiesis, cell proliferation, differentiation, and migration in some cells and organs. Numerous studies have focused on the expression and roles of FGFs in testis. FGFs are involved in the regulation of testicular development and maintaining germ cells functions in the normal and pathogenic conditions. FGFs promote testicular tumor development and metastasis. However, the underlying mechanism of FGFs roles in testis is not very clear and is required to be clarified. Understanding the role and mechanism of FGFs is the foundation in the discovery of treatments in testicular cancer, infertility, and diabetic complications.

## References

[1] Siu MKY, Cheng CY (2004). Dynamic cross-talk between cells and the extracellular matrix in the testis. *BioEssays*.

[2] Sánchez-Sánchez AV, Camp E, García-España A, Leal-Tassias A, Mullor JL (2010). Medaka Oct4 is expressed during early embryo development, and in primordial germ cells and adult gonads. *Developmental Dynamics*.

[3] Sakai Y, Suetake I, Itoh K, Mizugaki M, Tajima S, Yamashina S (2001). Expression of DNA methyltransferase (Dnmt1) in testicular germ cells during development of mouse embryo. *Cell Structure and Function*.

[4] Segretain D, Gilleron J, Carette D, Denizot J, Pointis G (2010). Differential time course of FSH/FSH receptor complex endocytosis within sertoli and germ cells during rat testis development. *Developmental Dynamics*.

[5] Krogenæs AK, Taubøll E, Stien A (2008). Valproate affects reproductive endocrine function, testis diameter and some semen variables in non-epileptic adolescent goat bucks. *Theriogenology*.

[6] Huhtaniemi I (1989). Endocrine function and regulation of the fetal and neonatal testis. *International Journal of Developmental Biology*.

[7] Li L, Donald JM, Golub MS (2005). Review on testicular development, structure, function, and regulation in common marmoset. *Birth Defects Research B*.

[8] Cotton LM, O’Bryan MK, Hinton BT (2008). Cellular signaling by fibroblast growth factors (FGFs) and their receptors (FGFRs) in male reproduction. *Endocrine Reviews*.

[9] Wagener A, Blottner S, Göritz F, Streich WJ, Fickel J (2003). Differential changes in expression of a and b FGF, IGF-1 and -2, and TGF-*α* during seasonal growth and involution of roe deer testis. *Growth Factors*.

[10] Hiramatsu R, Harikae K, Tsunekawa N, Kurohmaru M, Matsuo I, Kanai Y (2010). FGF signaling directs a center-to-pole expansion of tubulogenesis in mouse testis differentiation. *Development*.

[11] Mansukhani A, Bellosta P, Sahni M, Basilico C (2000). Signaling by fibroblast growth factors (FGF) and fibroblast growth factor receptor 2 (FGFR2)-activating mutations blocks mineralization and induces apoptosis in osteoblasts. *Journal of Cell Biology*.

[12] Medici D, Razzaque MS, DeLuca S (2008). FGF-23-Klotho signaling stimulates proliferation and prevents vitamin D-induced apoptosis. *Journal of Cell Biology*.

[13] Jiang X, Zhang C, Xin Y (2013). Protective effect of FGF21 on type 1 diabetes-induced testicular apoptotic cell death probably via both mitochondrial- and endoplasmic reticulum stress-dependent pathways in the mouse model. *Toxicology Letters*.

[14] Angelin B, Larsson TE, Rudling M (2012). Circulating fibroblast growth factors as metabolic regulators-a critical appraisal. *Cell Metabolism*.

[15] Kharitonenkov A (2009). FGFs and metabolism. *Current Opinion in Pharmacology*.

[16] Olsen SK, Garbi M, Zampieri N (2003). Fibroblast growth factor (FGF) homologous factors share structural but not functional homology with FGFs. *Journal of Biological Chemistry*.

[17] Ornitz DM (2001). Regulation of chondrocyte growth and differentiation by fibroblast growth factor receptor 3. *Novartis Foundation symposium*.

[18] Itoh N, Ornitz DM (2004). Evolution of the Fgf and Fgfr gene families. *Trends in Genetics*.

[19] Angelin B, Larsson TE, Rudling M (2012). Circulating fibroblast growth factors as metabolic regulators—a critical appraisal. *Cell Metabolism*.

[20] Itoh N (2010). Hormone-like (endocrine) Fgfs: their evolutionary history and roles in development, metabolism, and disease. *Cell and Tissue Research*.

[21] Chen L, Deng C (2005). Roles of FGF signaling in skeletal development and human genetic diseases. *Frontiers in Bioscience*.

[22] Marek L, Ware KE, Fritzsche A (2009). Fibroblast growth factor (FGF) and FGF receptor-mediated autocrine signaling in non-small-cell lung cancer cells. *Molecular Pharmacology*.

[23] Wu X, Ge H, Lemon B (2010). Separating mitogenic and metabolic activities of fibroblast growth factor 19 (FGF19). *Proceedings of the National Academy of Sciences of the United States of America*.

[24] Quarles LD (2012). Role of FGF23 in vitamin D and phosphate metabolism: implications in chronic kidney disease. *Experimental Cell Research*.

[25] Jameson SA, Lin YT, Capel B (2012). Testis development requires the repression of Wnt4 by Fgf signaling. *International Journal of Developmental Biology*.

[26] Gonzalez-Herrera IG, Prado-Lourenco L, Pileur F (2006). Testosterone regulates FGF-2 expression during testis maturation by an IRES-dependent translational mechanism. *The FASEB Journal*.

[27] Yamamoto H, Ochiya T, Takahama Y (2000). Detection of spatial localization of Hst-1/Fgf-4 gene expression in brain and testis from adult mice. *Oncogene*.

[28] Sprengel R, Braun T, Nikolics K, Segaloff DL, Seeburg PH (1990). The testicular receptor for follicle stimulating hormone: structure and functional expression of cloned cDNA. *Molecular Endocrinology*.

[29] Miller SC, Bowman BM, Rowland HG (1983). Structure, cytochemistry, endocytic activity, and immunoglobulin (Fc) receptors of rat testicular interstitial-tissue macrophages. *American Journal of Anatomy*.

[30] Steinberger E (1971). Hormonal control of mammalian spermatogenesis. *Physiological Reviews*.

[31] Enders GC, Kahsai TZ, Lian G, Funabiki K, Killen PD, Hudson BG (1995). Developmental changes in seminiferous tubule extracellular matrix components of the mouse testis: *α*3(IV) collagen chain expressed at the initiation of spermatogenesis. *Biology of Reproduction*.

[32] Siu MKY, Cheng CY (2008). Extracellular matrix and its role in spermatogenesis. *Advances in Experimental Medicine and Biology*.

[33] O’Shaughnessy PJ, Morris ID, Huhtaniemi I, Baker PJ, Abel MH (2009). Role of androgen and gonadotrophins in the development and function of the Sertoli cells and Leydig cells: data from mutant and genetically modified mice. *Molecular and Cellular Endocrinology*.

[34] Cancilla B, Risbridger GP (1998). Differential localization of fibroblast growth factor receptor-1, -2, -3, and -4 in fetal, immature, and adult rat testes. *Biology of Reproduction*.

[35] Lan Z, Labus JC, Hinton BT (1998). Regulation of gamma-glutamyl transpeptidase catalytic activity and protein level in the initial segment of the rat epididymis by testicular factors: role of basic fibroblast growth factor. *Biology of Reproduction*.

[36] Colvin JS, Green RP, Schmahl J, Capel B, Ornitz DM (2001). Male-to-female sex reversal in mice lacking fibroblast growth factor 9. *Cell*.

[37] Berta P, Hawkins JR, Sinclair AH (1990). Genetic evidence equating SRY and the testis-determining factor. *Nature*.

[38] Koopman P, Gubbay J, Vivian N, Goodfellow P, Lovell-Badge R (1991). Male development of chromosomally female mice transgenic for Sry. *Nature*.

[39] Kim Y, Kobayashi A, Sekido R (2006). Fgf9 and Wnt4 act as antagonistic signals to regulate mammalian sex determination. *PLoS Biology*.

[40] Cory AT, Boyer A, Pilon N, Lussier JG, Silversides DW (2007). Presumptive pre-Sertoli cells express genes involved in cell proliferation and cell signalling during a critical window in early testis differentiation. *Molecular Reproduction and Development*.

[41] Yamamoto S, Mikami T, Konishi M, Itoh N (2000). Stage-specific expression of a novel isoform of mouse FGF-14 (FHF-4) in spermatocytes. *Biochimica et Biophysica Acta*.

[42] Tagashira S, Harada H, Katsumata T, Itoh N, Nakatsuka M (1997). Cloning of mouse FGF10 and up-regulation of its gene expression during wound healing. *Gene*.

[43] Jaillard C, Chatelain PG, Saez JM (1987). In vitro regulation of pig Sertoli cell growth and function: effects of fibroblast growth factor and somatomedin-C. *Biology of Reproduction*.

[44] van Dissel-Emiliani FMF, de Boer-Brouwer M, de Rooij DG (1996). Effect of fibroblast growth factor-2 on Sertoli cells and gonocytes in coculture during the perinatal period. *Endocrinology*.

[45] Gonzalez A-M, Buscaglia M, Ong M, Baird A (1990). Distribution of basic fibroblast growth factor in the 18-day rat fetus: localization in the basement membranes of diverse tissues. *Journal of Cell Biology*.

[46] Han IS, Sylvester SR, Kim KH (1993). Basic fibroblast growth factor is a testicular germ cell product which may regulate Sertoli cell function. *Molecular Endocrinology*.

[47] Sordoillet C, Savona C, Chauvin MA (1992). Basic fibroblast growth factor enhances testosterone secretion in cultured porcine Leydig cells: site(s) of action. *Molecular and Cellular Endocrinology*.

[48] Laslett AL, McFarlane JR, Risbridger GP (1997). Developmental response by Leydig cells to acidic and basic fibroblast growth factor. *Journal of Steroid Biochemistry and Molecular Biology*.

[49] MacArthur CA, Shankar DB, Shackleford GM (1995). Fgf-8, activated by proviral insertion, cooperates with the Wnt-1 transgene in murine mammary tumorigenesis. *Journal of Virology*.

[50] Peters H (1970). Migration of gonocytes into the mammalian gonad and their differentiation. *Philosophical transactions of the Royal Society of London B*.

[51] Vergouwen RPFA, Jacobs SGPM, Huiskamp R, Davids JAG, de Rooij DG (1991). Proliferative activity of gonocytes, Sertoli cells and interstitial cells during testicular development in mice. *Journal of Reproduction and Fertility*.

[52] Valve E, Penttila T, Paranko J, Harkonen P (1997). FGF-8 is expressed during specific phases of rodent oocyte and spermatogonium development. *Biochemical and Biophysical Research Communications*.

[53] Kim Y, Bingham N, Sekido R, Parker KL, Lovell-Badge R, Capel B (2007). Fibroblast growth factor receptor 2 regulates proliferation and Sertoli differentiation during male sex determination. *Proceedings of the National Academy of Sciences of the United States of America*.

[54] Skinner MK, Norton JN, Mullaney BP, Rosselli M, Whaley PD, Anthony CT (1991). Cell-cell interactions and the regulation of testis function. *Annals of the New York Academy of Sciences*.

[55] Lu Q, Sun QY, Breitbart H, Chen D (1999). Expression and phosphorylation of mitogen-activated protein kinases during spermatogenesis and epididymal sperm maturation in mice. *Archives of Andrology*.

[56] Clermont Y, Leblond CP (1953). Renewal of spermatogonia in the rat. *The American Journal of Anatomy*.

[57] Cancilla B, Davies A, Ford-Perriss M, Risbridger GP (2000). Discrete cell- and stage-specific localisation of fibroblast growth factors and receptor expression during testis development. *Journal of Endocrinology*.

[58] Yamamoto H, Ochiya T, Tamamushi S (2002). HST-1/FGF-4 gene activation induces spermatogenesis and prevents adriamycin-induced testicular toxicity. *Oncogene*.

[59] Wagle A, Singh JP (2000). Fibroblast growth factor protects nitric oxide-induced apoptosis in neuronal SHSY-5Y cells. *Journal of Pharmacology and Experimental Therapeutics*.

[60] Vaahtokari A, Åberg T, Thesleff I (1996). Apoptosis in the developing tooth: association with an embryonic signaling center and suppression by EGF and FGF-4. *Development*.

[61] Hirai K, Sasaki H, Yamamoto H (2004). HST-1/FGF-4 protects male germ cells from apoptosis under heat-stress condition. *Experimental Cell Research*.

[62] Nishimura T, Nakatake Y, Konishi M, Itoh N (2000). Identification of a novel FGF, FGF-21, preferentially expressed in the liver. *Biochimica et Biophysica Acta*.

[63] Wente W, Efanov AM, Brenner M (2006). Fibroblast growth factor-21 improves pancreatic *β*-cell function and survival by activation of extracellular signal-regulated kinase 1/2 and Akt signaling pathways. *Diabetes*.

[64] Lü Y, Liu J, Zhang L (2010). Fibroblast growth factor 21 as a possible endogenous factor inhibits apoptosis in cardiac endothelial cells. *Chinese Medical Journal*.

[65] Tacer KF, Bookout AL, Ding X (2010). Research resource: comprehensive expression atlas of the fibroblast growth factor system in adult mouse. *Molecular Endocrinology*.

[66] Cai L, Chen S, Evans T, Deng DX, Mukherjee K, Chakrabarti S (2000). Apoptotic germ-cell death and testicular damage in experimental diabetes: prevention by endothelin antagonism. *Urological Research*.

[67] Zhao Y, Tan Y, Dai J (2011). Exacerbation of diabetes-induced testicular apoptosis by zinc deficiency is most likely associated with oxidative stress, p38 MAPK activation, and p53 activation in mice. *Toxicology Letters*.

[68] Xiao J, Lv Y, Lin S (2010). Cardiac protection by basic fibroblast growth factor from ischemia/reperfusion-induced injury in diabetic rats. *Biological and Pharmaceutical Bulletin*.

[69] Cronauer MV, Schulz WA, Seifert H-H, Ackermann R, Burchardt M (2003). Fibroblast growth factors and their receptors in urological cancers: basic research and clinical implications. *European Urology*.

[70] Suzuki K, Tokue A, Kamiakito T, Kuriki K, Saito K, Tanaka A (2001). Predominant expression of fibroblast growth factor (FGF) 8, FGF4, and FGF receptor 1 in nonseminomatous and highly proliferative components of testicular germ cell tumors. *Virchows Archiv*.

[71] Strohmeyer T, Peter S, Hartmann M (1991). Expression of the hst-1 and c-kit protooncogenes in human testicular germ cell tumors. *Cancer Research*.

[72] Yoshida T, Tsutsumi M, Sakamoto H (1988). Expression of the HST1 oncogene in human germ cell tumors. *Biochemical and Biophysical Research Communications*.

[73] Granerus M, Welin A, Lundh B, Schofield PN, Ekstrom TJ, Engstrom W (1993). Heparin binding growth factors and the control of teratoma cell proliferation. *European Urology*.

[74] Lau MT, So WK, Leung PC (2013). Fibroblast growth factor 2 induces E-cadherin down-regulation via PI3K/Akt/mTOR and MAPK/ERK signaling in ovarian cancer cells. *PloS ONE*.

[75] Lefèvre G, Babchia N, Calipel A (2009). Activation of the FGF2/FGFR1 autocrine loop for cell proliferation and survival in uveal melanoma cells. *Investigative Ophthalmology and Visual Science*.

[76] Matsuo M, Yamada S, Koizumi K, Sakurai H, Saiki I (2007). Tumour-derived fibroblast growth factor-2 exerts lymphangiogenic effects through Akt/mTOR/p70S6kinase pathway in rat lymphatic endothelial cells. *European Journal of Cancer*.

[77] Peng C, Pan S, Lee K, Bastow KF, Teng C (2008). Molecular mechanism of the inhibitory effect of KS-5 on bFGF-induced angiogenesis in vitro and in vivo. *Cancer Letters*.

[78] Wu J, Yan H, Chen W (2008). JNK signaling pathway is required for bFGF-mediated surface cadherin downregulation on HUVEC. *Experimental Cell Research*.

[79] Partridge CR, Hawker JR, Forough R (2000). Overexpression of a secretory form of FGF-1 promotes MMP-1-mediated endothelial cell migration. *Journal of Cellular Biochemistry*.

[80] Lungu G, Covaleda L, Mendes O, Martini-Stoica H, Stoica G (2008). FGF-1-induced matrix metalloproteinase-9 expression in breast cancer cells is mediated by increased activities of NF-kappa;B and activating protein-1. *Molecular Carcinogenesis*.

[81] Aigner A, Brachmann P, Beyer J (2003). Marked increase of the growth factors pleiotrophin and fibroblast growth factor-2 in serum of testicular cancer patients. *Annals of Oncology*.

[82] Kelleher FC, O'Sullivan H, Smyth E (2013). Fibroblast growth factor receptors, developmental corruption and malignant disease. *Carcinogenesis*.

[83] Chou T, Finn RS (2012). Brivanib: a review of development. *Future Oncology*.

[84] Arai Y, Marui A, Komeda M (2006). Regenerative medicine with the sustained release system of basic fibroblast growth factor. *Nippon Rinsho*.

[85] Hinton BT, Lan ZJ, Rudolph DB, Labus JC, Lye RJ (1998). Testicular regulation of epididymal gene expression. *Journal of reproduction and fertility. Supplement*.

